# Germination ecology of *Chenopodium album* L. and implications for weed management

**DOI:** 10.1371/journal.pone.0276176

**Published:** 2022-10-17

**Authors:** Wei Tang, Haipeng Guo, Jianing Yin, Xiaohui Ding, Xiaoyan Xu, Tingru Wang, Chao Yang, Wangdan Xiong, Shangzhi Zhong, Qibo Tao, Juan Sun

**Affiliations:** 1 College of Grassland Science, Qingdao Agricultural University, Qingdao, China; 2 Key Laboratory of National Forestry and Grassland Administration on Grassland Resources and Ecology in the Yellow River Delta, College of Grassland Science, Qingdao Agricultural University, Qingdao, China; New South Wales Department of Primary Industries, AUSTRALIA

## Abstract

*Chenopodium album* L. is a troublesome annual species in various cropping systems, and a sound knowledge of the ecological response of *C*. *album* germination to environmental factors would suggest suitable management strategies for inhibiting its spread. Preliminary laboratory-based research was conducted to investigate germination and emergence requirements of *C*. *album* under various environmental conditions (e.g., photoperiods, constant temperature, salinity, moisture, soil pH, burial depth, and oat crop residue). Results showed *C*. *album* seeds were found to be photoblastic, with only 13% germination in darkness. The maximum germination (94%) of *C*. *album* occurred at an optimal temperature of 25°C, and the depressive effect of other temperatures on germination was more severe at lower rather than higher temperatures. Seed germination was suitably tolerant of salinity and osmotic potential, with germination observed at 200 mM NaCl (37.0%) and -0.8 MPa (20%), respectively. Germination was relatively uniform (88–92%) at pH levels ranging from 4 to 10. The maximum germination of *C*. *album* was observed on the soil surface, with no or rare emergence of seeds at a burial depth of 2 cm or under 7000 kg ha^-1^ oat straw cover, respectively. Information provided by this study will help to develop more sustainable and effective integrated weed management strategies for the control of *C*. *album*, including (i) a shallow-tillage procedures to bury weed seeds in conventional-tillage systems and (ii) oat residue retention or coverage on the soil surface in no-tillage systems.

## Introduction

Globally, weeds are one of the greatest biotic threats to sustainable crop production. Integrated weed management (IWM) strategies are more reliable and effective in cropping systems, enabling farmers to minimize the negative impact of herbicides and maintain high crop yield [1]. Development of IWM depends on a sound knowledge of a weed’s biology and ecology. Seed germination and seedling emergence are critical stages in most weed lifecycles and thus determine the successful survival, establishment, and dispersal of weeds in an agro-environment [2]. Therefore, a comprehensive knowledge of seed germination traits is essential to develop strategies for successful management and/or suppression of weed seed germination. In this regard, information is needed on the longevity of weed seeds in the soil, the abundance and timing of germination in relation to the germination of crop species, and the development of herbicide resistance. Germination and seedling emergence are affected not only by seed properties but also by various external factors, such as temperature, salinity, pH, soil moisture, burial depth, and agronomic management practices [3, 4].

*Chenopodium album* L. is one of the world’s most noxious weeds, and it prefers sub-tropical, tropical, and temperate regions of the world [1, 5]. The species is invasive in China and recognized as one of the most widespread and problematic weed species in the industrialization of cropping systems, including under conventional tillage coupled with intensive use of herbicides and nitrogen fertilizers [6]. It often emerges throughout the spring and summer, with the peak of germination occurring in early to mid-spring and rapidly infests agricultural fields [7, 8]. While *C*. *album* is a self-pollinated species, some cross-pollination by wind also occurs in the species [9]. A huge contributor to the rapid spread of this species is its high seed production of more than 70,000 mature seeds per plant, and seeds can remain viable in the soil for 30 to 40 years [10]. In this respect, high seed production facilitates it building up rich and high-longevity seedbanks, thereby promoting its population persistence and new infestations in fields [9]. Furthermore, a proportion of *C*. *album* seeds become permeable soon after seed dispersal, which means they are therefore able to germinate when conditions are favorable, while other seeds will break their dormancy after winter, after which, by next spring, they can germinate at normal habitat temperatures, thereby imposing a serious and conterminous problem throughout the crop growing season [11, 12]. These unique features ensure that *C*. *album* poses severe competition with crops from the early growth stages onward, resulting in great reductions in both crop yield and quality [13].

*C*. *album* has been reported to grow naturally as a highly competitive species among crops, being particularly aggressive in fields of soybean (*Glycine max* (L.) Merr.), rapeseed (*Brassica napus* L.), maize (*Zea mays* L.), and wheat (*Triticum aestivum* L.) in various geographic regions of the world [14, 15]. Maize yield is reduced more by *C*. *album* than by many other weeds (e.g., smartweed (*Polygonum lapathifolium* L.) and powell’s amaranth (*Amaranthus powellii* L.)) [16]. There is a critical period of weed competition in maize, as maize yield decreased by more than 70% when *C*. *album* emerged 7 and 14 d earlier at high density (16 and 20 plants m^-2^) [17]. The economic threshold, six *C*. *album* plants m^-2^, would help in making decisions for *C*. *album* management in wheat field [18]. In addition, *C*. *album* reduces the yield of horticultural crops. Hwang et al. [19] indicated *C*. *album* is the most dominant weed species in both onion (*Allium cepa* L.) and Chinese cabbage (*Brassica pekinensis* (Lour.) Rupr.) fields in Korea. Similarly, a flower yield reduction of 42% was recorded in marigold (*Calendula officinalis* L.) with two *C*. *album* plants m^-2^ in Iran [20]. Therefore, effective management strategies are necessary for *C*. *album* control.

An accurate understanding of the environmental factors affecting the germination and emergence of agricultural weed populations can be crucial in achieving successful control management in an agroecosystem. Accordingly, much research has been conducted on *C*. *album* in many countries; thus, much information is currently available in the literature about its seed the dormancy-breaking and germination requirements. For example, the dormancy of black *C*. *album* seeds can be alleviated either after storage for 330 d at room temperature [21] or after ripening at low temperatures in the dark moist conditions for 4 to 52 weeks [8, 22, 23]. High levels of endogenous nitrate can improve the germination response to ethylene or GA4+7 [24], and exposure of seeds to exogenous nitrate can promote germination [22, 25]. Additionally, seeds of *C*. *album* are photoblastic and have strict light requirement for germination. *C*. *album* seed germination was positively affected by increased photoperiod length, whereas decreased photoperiod length maintained a high dormancy percentage of seeds [8, 26, 27]. The seed germination of *C*. *album* may occur across a wide range of temperatures (5–30°C) [13, 28], and alternating temperatures resulted in seeds that germinated readily at 20–30°C or 10–30°C [26]. *C*. *album* was sensitive to osmotic stress, and no germination occurred at −0.6 MPa within the temperature range of 7.5–42.5°C [29]. The 300 mM salinity induced a 50% reduction in germination for the *C*. *album* seeds [30]. Small *C*. *album* seeds were delayed or unable to emerge from below 2 cm burial depths [21]. Although, some information is available on *C*. *album* seed germination, a complete understanding of the impact of different environmental factors on seed germination of *C*. *album* is lacking. Detailed information on the ecological requirements of *C*. *album* seed germination characterizes its germination niche and the habitats in which it is likely to germinate and emerge [7]. In addition, within agroecosystems, crop residues (e.g., straw mulch) can create a physical barrier and a shading microenvironment for weed suppression in IWM programs [4]. Before the adoption of reduced- or no-till systems, crop residues were normally burned or buried though deep ploughing in conventional tillage, whereas the widespread adoption of conservation agriculture systems globally has facilitated stubble retention in IWM programs [31]. Oat (*Avena sativa* L.) is a major grain and forage crop, and its stubble (3000 kg ha^-1^−8000 kg ha^-1^) is often left in the field after harvest, with oat straw mulch exhibiting a high weed suppression ability [32]. However, information is also scant regarding the emergence behavior of *C*. *album* under oat residue cover.

A thorough synthesis of the available information on the germination ecology of *C*. *album* could be utilized to develop a management plan for *C*. *album* when intervention can be cost-effective and when its seeds are environmentally sensitive. The present study aimed to systematically evaluate and compare the germination and emergence abilities of *C*. *album* in response to the environmental factors of light conditions, constant temperature, salinity, osmotic stress, pH, soil burial depth, and oat residue cover. The results of the current study can be used to assist management of *C*. *album* in native and introduced agro-ecological systems, through science-based prediction, prevention, and control of this species.

## Materials and methods

### Seed collection

Mature *Chenopodium album* seeds were collected from plants in a forage field that had been cultivated with alfalfa for two years in the Jiaozhou District of Shandong Province, China (36°26′25″N, 120°4′48″E; elevation, 1 m) during the start of the summer season in July 2021. Mature *C*. *album* seeds, which were characterized by their dark black coloration, were collected from 50–60 mother plants after the leaves and stems had yellowed [22]. After their collection, seeds were maintained properly enwrapped by the pericarp and perianth and dried in the shade at room temperature (20–25°C) for seven days (with no after-ripening treatment) prior to being stored in paper bags. Seeds visibly damaged by insects or pathogens were discarded. Dormancy of *C*. *album* mature seeds was about 73.5%. Then, *C*. *album* seeds were stored in cold storage at -20°C for 6 months as a dormancy-breaking treatment [33].

### General seed germination protocol

At the beginning of the experiment, seeds were removed from -20°C storage. Germination tests revealed more than 90% germination of *C*. *album* seeds, indicating dormancy had indeed been broken. Before the commencement of subsequent germination or seedling emergence tests, seeds were surface sterilized by been soaked in 1% sodium hypochlorite (NaClO) for 5 min and rinsed five times with distilled water. Four replications of 50 seeds of *C*. *album* were placed in a 9-cm-diameter Petri dish lined with two layers of sterile Whatman^®^ No. 11 filter paper (Whatman, Maidstone, UK), moistened with either 5 ml of distilled water or a treatment solution (for photoperiod, temperature, salinity, osmotic potential and pH experiments) and sealed with Parafilm^®^ (Bemis Company, Neenah, WI, USA) to ensure moisture retention. The Petri dishes were placed in incubators (GXZ-380B, Jiangnan Instrument Manufacture, Vol. 380 L, Ningbo, China) equipped with cool-white fluorescent tubes with a photosynthetic photon flux of 100 μmol m^-2^ s^-1^, set to a 12-h alternating light/dark cycle. Germination tests were conducted for four weeks, and the criterion for germination was visible protrusion of the radicle having emerged by 2-mm. Germinated seeds were counted and removed daily from dishes. Based on the findings of the photoperiod and temperature experiment, all subsequent seed germination experiments were conducted at 25°C and under a 12-h photoperiod. All experiments were terminated after four weeks, and repeated over time (two experimental runs for each treatment). The experiments were laid out according to a randomized complete block design.

### Effect of photoperiod on germination

Seeds were exposed to continuous dark and alternating light/dark (12 h/12 h and 8 h/16 h) regimes in incubators. The dark treatment involved wrapping each dish in four layers of aluminum foil immediately after sowing. To prevent exposure of seeds to white light during the germination trials, Petri dishes assigned to 24-h dark treatment were opened under a safe green light. A constant day/night temperature of 25°C was maintained for all the tests. For each photoperiod, four replications of 50 seeds per Petri dish were used. The selection of this photoperiod was based on results of Alshallash et al. [26], who showed photoperiod effects on seed germination of *C*. *album*. All other experimental conditions were the same as described in the general seed germination protocol.

### Effect of temperature on germination

To test the effect of temperature on seed germination, *C*. *album* seeds were incubated under 9 different constant-temperature regimes (10°C, 15°C, 18°C, 20°C, 25°C, 30°C, 35°C, 38°C, and 40°C) under a 12-h photoperiod. For each temperature, four replications of 50 seeds per Petri dish were used. The temperature range selected mostly corresponded to the temperature variation from spring to autumn of the seed collection habitat and the surrounding region that the species might experience. All other experimental conditions were the same as described in the general seed germination protocol.

### Effect of salt stress on germination

A range of concentrations of sodium chloride (NaCl) solutions was used to test the influence of salinity on *C*. *album* seed germination. The concentrations used were 0 (distilled water for the control), 50, 100, 150, 200, 250, and 300 mM NaCl. For each salinity level, four replications of 50 seeds per Petri dish were used. These concentrations reflect the range of salinity soil conditions and ground water used for irrigation in China [34, 35]. All other experimental conditions were the same as described in the general seed germination protocol.

### Effect of osmotic potential on germination

Effects of osmotic stress on *C*. *album* seed germination were tested in aqueous polyethylene glycol 6000 (PEG) solutions (Sigma Aldrich, St Louis,MO, USA). PEG-6000 was dissolved in distilled water to develop solutions of various respective osmotic potentials (0, −0.1, −0.2, −0.4, −0.6, −0.8, −1.0 and −1.2 MPa) for the germination assay [36]. For each osmotic potential, four replications of 50 seeds per Petri dish were used. All other experimental conditions were the same as described in the general seed germination protocol.

### Effect of pH on germination

The effects of variation in pH on seed germination were tested by germinating the seeds in buffer solutions ranging from pH 4 to 10, which are values representative of soils in China. To achieve the desired level of pH, buffer solutions were prepared following Chadha et al. [37]. A 2 mM solution of 2-(*N*-morpholino) ethanesulfonic acid (MES) was used to prepare solutions with pH values of 4, 5, and 6 with an aqueous form of 1 N NaOH. Similarly, a 2 mM solution of *N*-(2-hydroxymethyl) piperazine *N*-(2-ethane sulfonic acid) (HEPES) was used to prepare solutions with pH values of 7 and 8 with 1 N NaOH. The solutions with pH values of 9 and 10 were prepared with 2 mM N-tris (hydroxymethyl) methyl glycine (tricine) and 1 N NaOH. For each pH, four replications of 50 seeds per Petri dish were used. All other experimental conditions were the same as described in the general seed germination protocol.

### Burial depth on seedling emergence

To evaluate the effect of different burial depths on *C*. *album* seedling emergence, seeds were buried at 0 cm (i.e., placed on the soil surface), 0.1, 0.2, 0.3, 0.4, 0.5, 1, and 2 cm depths of soil in pot experiment. For each burial depth, four replications of 50 seeds per pot were used. The soil was collected from the seed collection site. The soil was brown earth with a pH of 7.57, and the organic C, total N, and total P were 16.96, 0.88, and 0.45 g kg^-1^, respectively. Available N and P were 77.12 and 27.05 mg kg^-1^, respectively. The soil was passed through a 3-mm sieve and autoclaved before the experiment to ensure that there were no living seeds in the soil. Specifically, the soil was loaded into polyethylene autoclave bags, which were placed into the autoclave. Then, the soil was processed using in the “solid” cycle (120°C for 50 minutes with both pre- and post-cycle vacuum stages) for sterilization. Each pot (14 cm diameter, 15 cm height) was filled with sterilized soil (1000g; soil water content, 23.6%), and *C*. *album* seeds were scattered onto the soil surface and covered with soil to achieve the various specified depths. A millimeter ruler was used to ensure, as accurately as possible, that all seeds in a replicate were buried at the appropriate depths. The seeds were incubated under constant temperatures of 25°C and 12 h light/ 12 h dark in the incubator, and generally the plot size was 380 L. Seedlings were considered to be ‘emerged’ when the cotyledon was visible at the soil surface. Seedling emergence was recorded daily for four weeks or until emergence no longer occurred, if any had emerged, but seedlings were not removed so as to prevent any possible disturbance of the soil. Throughout the experiment, pots were watered every other day with a mist sprinkler to maintain adequate soil moisture. The experiment was terminated after four weeks and repeated over time (two experimental runs for each treatment). The experiment was laid out according to a randomized complete block design.

### Effect of mulch cover on seedling emergence

The effect of oat mulch cover treatments of 0, 2000, 3000, 4000, 5000, 6000, and 7000 kg ha^-1^ on seedling emergence of *C*. *album* was evaluated in pot experiment. For each mulch cover treatment, four replications of 50 seeds per pot were used. Oat residues left after harvesting were applied as soil mulching. The oat mulch cover was prepared using a modified method described by Zangoueinejad and Alebrahim [38]. Oat straw (leaves and stems) were dried and chopped in a grinding mill to prepare mulch. Each plastic pot (14 cm diameter, 15 cm height) was filled with sterilized soil, the same soil as described previously for the seed burial depth experiment. The seeds were placed at the soil surface which obtained the highest germination percentage, and the resultant powder of oat straw was spread on the soil surface at a rate equivalent to the specified levels of the treatments. The amount of oat residue (as straw mulch) used in this study was representative of the quantity of mulch levels after harvest found in irrigated and dry land farming systems in China [39, 40]. The trials were incubated under constant temperatures of 25°C and 12 h light/ 12 h dark in the incubator, and generally the plot size was 380 L. Seedling emergence, i.e., when the cotyledon was visible above the soil surface, was counted daily for four weeks or until emergence no longer occurred. If any had emerged, seedlings were not removed to avoid any possible disturbance of the soil and cover. Pots were watered every other day to maintain adequate soil moisture levels. The experiment was terminated after four weeks, and repeated over time (two experimental runs for each treatment). The experiment was laid out according to a randomized complete block design.

### Statistical analyses

All experiments were arranged in a completely randomized design with four replicates. Experiments were repeated over time, and the second run of experiments was started within a week of the completion of the first run. Datasets from repeated experiments were subjected to analysis of variance (ANOVA) using SPSS software (v. 17.0, SPSS Inc., Chicago, IL, USA), which indicated no statistically significant run-by-treatment interaction for any experiment. The data from all eight replicates were therefore pooled across runs and used for subsequent analyses. Prior to conducting the ANOVA, the assumptions of normality and homogeneity of variance were investigated. Data were arcsine-transformed or log-transformed when the normality of the residuals was rejected by a Shapiro–Wilk test, while homoscedasticity of the variables was unequal according to Levene’s test. The data transformations used prior to the ANOVA ensured the transformed data satisfied the assumptions of normality and homogeneity of variance. When the F-test indicated statistical significance at *P* < 0.05, Duncan’s new multiple range test for least significant difference (LSD) was used to determine the least significant range between means.

For each parameter, germination or emergence percentage (GP or EP), mean germination or emergence time (MGT or MET), and germination or emergence index (GI or EI) were evaluated.

The GP or EP was calculated using the formula GP or EP = (*a*/*b*) × 100%, where *a* is the total number of seeds germinated or seedlings that have emerged and *b* is the total number of seeds tested.

MGT or MET is a measure of the rate and time-spread of germination or emergence, and it was calculated as:

$$MGT\;(MET)=\sum (D_{n})/\sum n.$$ (1)

where *n* is the number of germinated seeds or emerged seedlings on day *D* and *D*_n_ indicates the number of days from the beginning of germination/emergence.

The GI or EI is the sum of the ratios of the un-germinated seeds or un-emerged seedlings from the day of the first count until the final day of counting and was recorded as defined by applying the following formula:

GI(EI)=No.ofgerminatedseeds(emergedseedling)/Daysoffirstcount+…..+No.ofgerminatedseeds(emergedseedling)/Daysoffinalcount. (2)

The GP or EP data collected were modelled using two different types of models: logistic and Gaussian. SigmaPlot (Version 11, Sysat Software, Inc., Point Richmond, CA, USA) was used to perform nonlinear regression analysis to determine how seed germination or seedling emergence was affected by temperature, salinity level, osmotic potential, burial depth and mulch cover. The goodness-of-fit of models was assessed according to their *R*^2^ and root mean-square error (RMSE) values.

Nonlinear regression analysis was applied to calculate the effect of NaCl, osmotic stress, burial depth, and mulch cover on the seed germination (seedling emergence) percentage following analysis with the following functional three-parameter logistic model:

$$Y=A/[1+{\left(x/x_{50}\right)}^{B}].$$ (3)

Here, *Y* is the percentage (%) of seed germination or emergence under NaCl, osmotic potential, sowing depth, and mulch cover treatments, *A* is the maximum germination or emergence (%), *x*_50_ is the salinity level, osmotic potential, sowing depth, and mulch cover required for 50% suppression of the highest germination or emergence, and *B* represents the slope.

Similarly, the germination (%) at different temperature ranges were fitted using a three-parameter Gaussian model:

$$Y=A*e[‐0.5*{\left\{(x‐B)/C\right\}}^{2}].$$ (4)

Here, *A* represents the highest percentage (%) of seed germination, *B* represents the respective environmental condition that achieved the highest germination, and *C* represents the variance of the Gaussian distribution.

## Results

### Photoperiod

**Fig 1:**
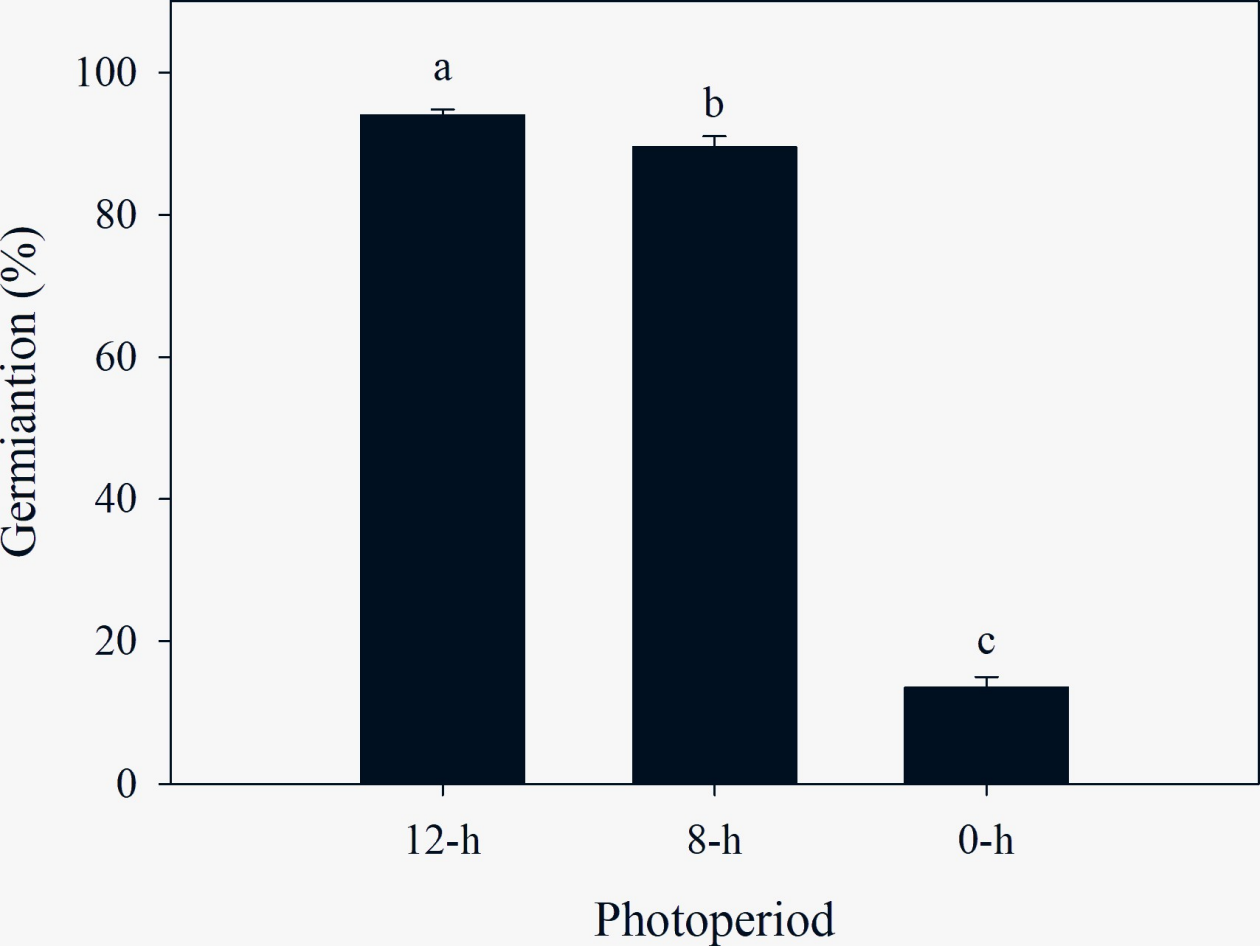
Effect of different light/dark regimes on germination percentage of *C*. *album*. Data represent the mean ± standard error of the mean (*n* = 8).

Seed germination was influenced by photoperiod. The seeds had a strict light requirement for germination, i.e., germination was rarely recorded under dark conditions. Moreover, a higher seed germination percentage was recorded under a 12-h photoperiod compared to an 8-h photoperiod (Fig 1). Furthermore, the mean time of germination (MTG) and germination index (GI) of *C*. *album* was significantly affected by temperature (Table 1). Earlier MTG was observed under a 12-h photoperiod compared to an 8-h photoperiod. The GI of *C*. *album* was at its maximum under a 12-h photoperiod, whereas the minimum GI was observed under a fully dark treatment (Table 1).

**Table 1:** Effect of photoperiods on germination traits of *C*. *album*.

Photoperiods (h)	Mean germination time (MGT) (days)		Germination index (GI)
12	3.82ab (0.03)		12.90a (0.06)
8	4.10a (0.13)		11.45b (0.43)
0	3.48b (0.15)		2.07c (0.20)
Source	D.F.	MGT	GI
Photoperiods	2	0.013*	<0.001***

Note: All values are showed as mean (S.E.) (*n* = 8). Within a column for MGT and GI of each temperature, values followed by different letters are significantly different by least significant differences of Duncan’s new multiple range test.

* and *** mean *P* < 0.05 and *P* < 0.001, respectively.

### Constant temperatures

**Fig 2:**
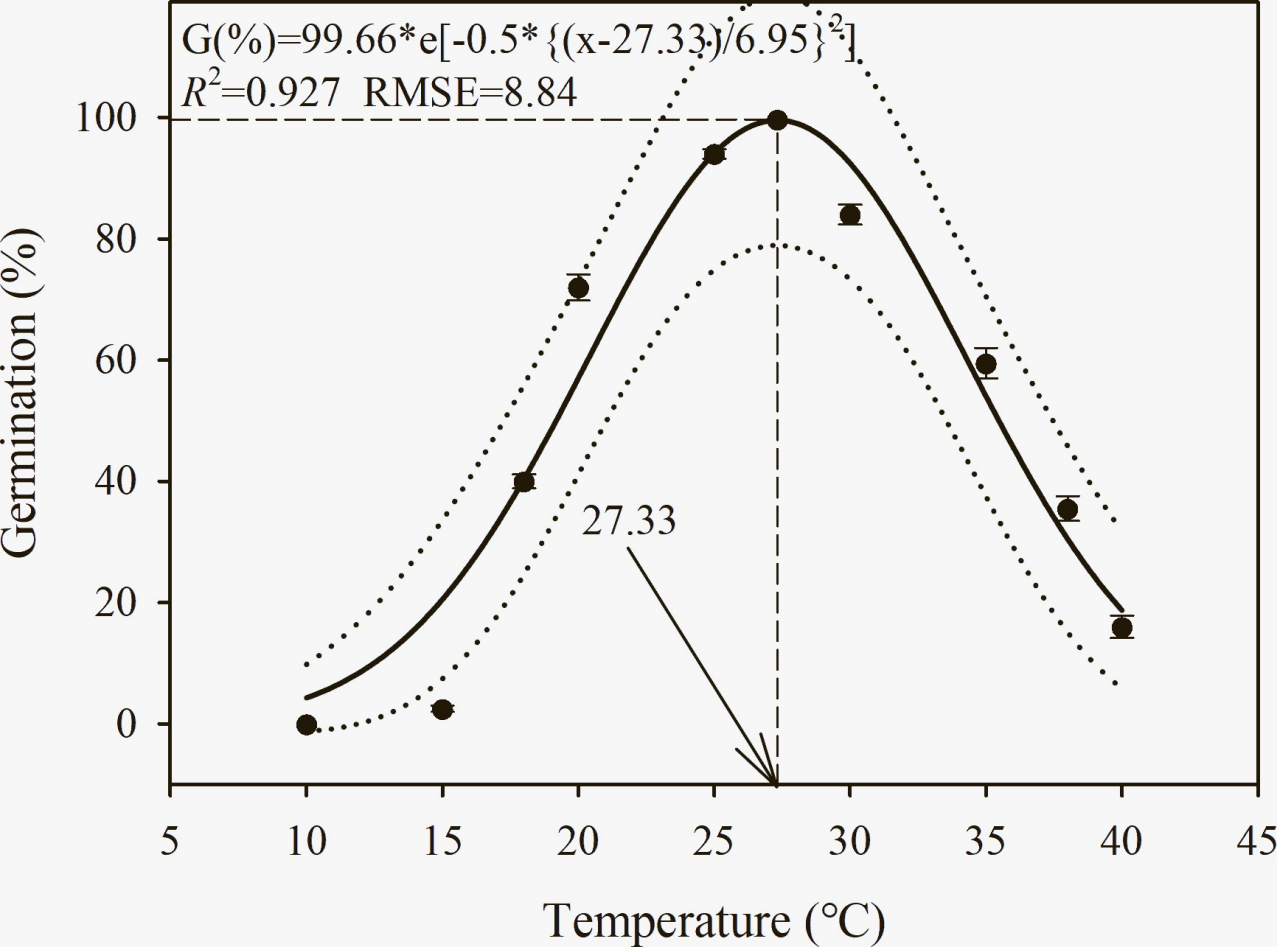
Effect of constant temperatures (°C) on germination percentage of *C*. *album*. The solid lines represent a three-parameter Gaussian model fitted to the data of *C*. *album* and thin dotted lines show 95% confidence intervals. G (%) = 99.66*e[-0.5*{(x—27.33)/6.95}^2^], *R*^2^ = 0.927, RMSE = 8.84. Data represent the mean ± standard error of the mean (*n* = 8).

A three-parameter Gaussian model {G (%) = 99.66*e [-0.5*{(*x*—27.33)/6.95}^2^], *R*^2^ = 0.927, RMSE = 8.84} was fitted to assess the seed germination percentage of *C*. *album* at different temperatures (Fig 2). Germination of *C*. *album* seeds occurred between 15°C and 40°C. An increase in seed germination percentage was recorded as the temperature rose up to 25°C and then sharply declined. The maximum germination (94%) was recorded at 25°C followed by 30°C (84%). The minimum germination (2.5%) was attained at the lowest temperature (15°C). According to the model, the highest germination occurred at a temperature of 27.3°C (Fig 2).

The MTG and GI of *C*. *album* seed germination was significantly affected by temperature (Table 2). The maximum mean time of germination (MTG) was observed at 18°C (8 days), while the minimum time was observed at 25°C and 30°C (3.4 and 3.9 d, respectively). The germination index (GI) of *C*. *album* was at its maximum (12.9 and 13.0) at 25°C and 30°C, respectively, while the minimum GI (0.25) was observed at 15°C (Table 2).

**Table 2:** Effect of temperature on germination traits of *C*. *album*.

Temperature (T) (°C)	Mean germination time (MGT) (days)		Germination index (GI)
10	-^a^		-^a^
15	5.00c (0.00)		0.25f (0.05)
18	7.92a (0.34)		2.80d (0.14)
20	6.43b (0.19)		6.31c (0.17)
25	3.82d (0.03)		12.90a (0.06)
30	3.42d (0.04)		13.00a (0.40)
35	2.33e (0.08)		9.87b (0.42)
38	3.20d (0.21)		6.32c (0.45)
40	2.53e (0.43)		1.80e (0.25)
Source	D.F.	MGT	GI
T	7	< 0.001***	< 0.001***

Note: All values are showed as mean (S.E.) (*n* = 8). Within a column for MGT and GI of each temperature, values followed by different letters are significantly different by least significant differences of Duncan’s new multiple range test.

*** means *P* < 0.001.

^a^, seed were no germination at 10°C; thus, MGT and GI could not be calculated.

### Salt stress

**Fig 3:**
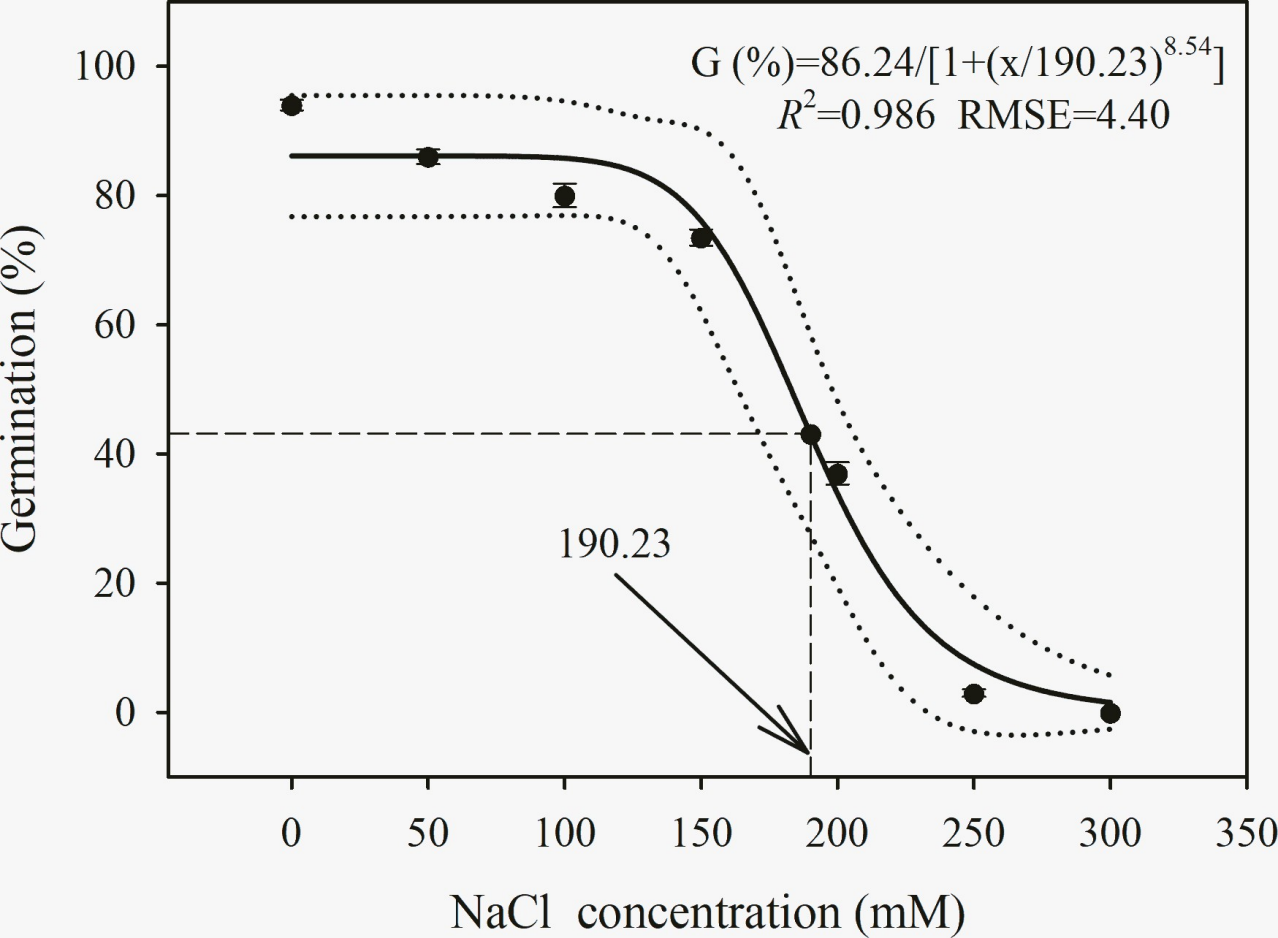
Effect of NaCl concentrations (mM) on germination percentage of *C*. *album*. The solid lines represent a three-parameter logistic model fitted to the data of *C*. *album* and thin dotted lines show 95% confidence intervals. G (%) = 86.2/[1+ (x/190.2)^8.5^], *R*^2^ = 0.986, RMSE = 4.40. Data represent the mean ± standard error of the mean (*n* = 8).

A three-parameter logistic model {*G* (%) = 86.2/[1+ (*x*/190.2)^8.5^], *R*^2^ = 0.986, RMSE = 4.40} was used to model the ability of *C*. *album* to germinate at various NaCl concentrations (Fig 3). The seed germination of *C*. *album* slowly decreased to a concentration of 150 mM NaCl and then sharply declined. *C*. *album* seed germination was 73.5% under 150 mM NaCl, and the lowest germination (3.0%) was measured under 250 mM NaCl. According to the model, a 50% reduction from the maximum germination percentage was achieved at 190.2 mM of NaCl (Fig 3).

The MTG and GI values of *C*. *album* seed germination were significantly affected by salinity (Table 3). The maximum MGT (10 d) of *C*. *album* was recorded at 250 mM NaCl, and a decreased in MGT was recorded under a low level of NaCl stress. The maximum GI (12.9) was recorded under 0 mM NaCl, followed by those GI values observed under 50 and 100 mM NaCl. The lowest GI (0.15) was recorded under 250 mM NaCl (Table 3).

**Table 3:** Effect of salinity levels on germination traits of *C*. *album*.

NaCl concentration (NaCl) (mM)	Mean germination time (MGT) (days)		Germination index (GI)
0	3.82e (0.03)		12.90a (0.06)
50	4.81d (0.09)		9.53b (0.22)
100	6.01c (0.31)		7.18c (0.36)
150	8.07b (0.27)		5.03d (0.15)
200	8.40b (0.23)		2.41e (0.13)
250	10.00a (0.35)		0.15f (0.02)
300	-		-^a^
Source	D.F.	MGT	GI
NaCl	5	< 0.001***	< 0.001***

Note: All values are showed as mean (S.E.) (*n* = 8). Within a column for MGT and GI of each temperature, values followed by different letters are significantly different by least significant differences of Duncan’s new multiple range test.

*** means *P* < 0.001.

^a^, seed were no germination at 300 mM; thus, MGT and GI could not be calculated.

### Osmotic stress

**Fig 4:**
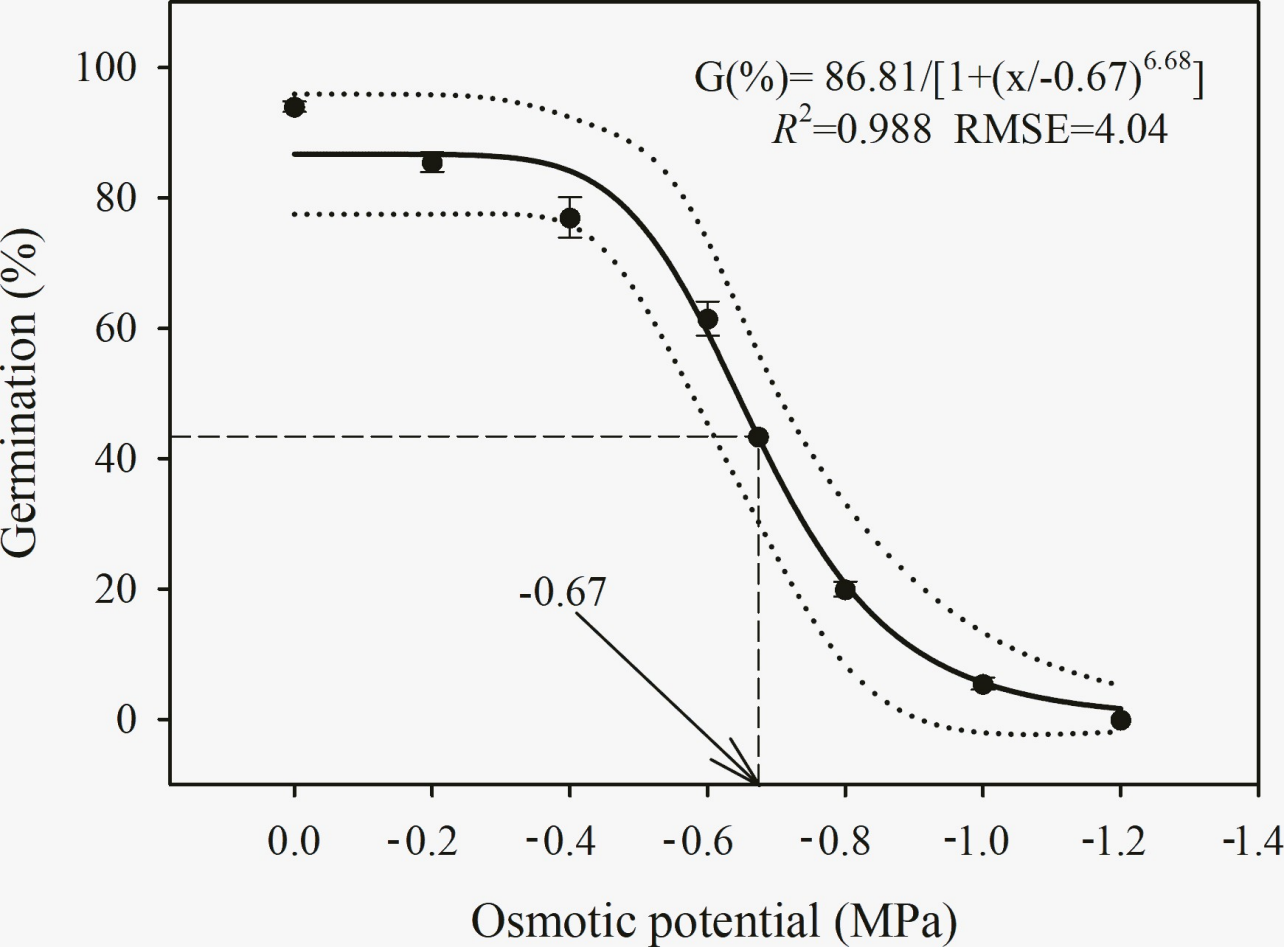
Effect of osmotic potentials (MPa) on germination percentage of *C*. *album*. The solid lines represent a three-parameter logistic model fitted to the data of *C*. *album* and thin dotted lines show 95% confidence intervals. G (%) = 86.8/[1 + (x/-0.67)^6.7^], *R*^2^ = 0.988, RMSE = 4.04. Data represent the mean ± standard error of the mean (*n* = 8).

A three-parameter logistic model {G (%) = 86.8/[1 + (*x*/-0.67)^6.7^], *R*^2^ = 0.988, RMSE = 4.04} was used to model the ability of *C*. *album* to germinate at various water potentials (Fig 4). Germination was reduced from 94% to 5.5% as osmotic stress increased from 0 to −1.0 MPa. Similar levels of seed germination were observed in *C*. *album* up to −0.4 MPa, and subsequently, a dramatic decrease in seed germination was observed at lower water potentials (−0.6 to −1.0 MPa). At −1.2 MPa, seed germination was completely suppressed. According to the model, a 50% reduction from the maximum germination rate was achieved at −0.67 MPa (Fig 4).

The MTG and GI of *C*. *album* seed germination was significantly affected by osmotic potential (Table 4). The maximum MGT was observed at −1.0 MPa (14 d), and the minimum MGT was observed at 0 MPa (3.8 d). On average, the adverse effect of osmatic stress on MGT was more pronounced (*P* < 0.05) at lower water potentials (−0.6 and −0.8 MPa) relative to higher water potentials (−0.2 and −0.4 MPa). In the control treatment (0 MPa), the maximum GI was recorded (13.0), while the minimum GI (0.2) was observed at −1.0 MPa (Table 4).

**Table 4:** Effect of osmotic potential on germination traits of *C*. *album*.

Osmotic potential (OP) (MPa)	Mean germination time (MGT) (days)		Germination index (GI)
0	3.82e (0.03)		12.90a (0.06)
-0.2	5.18d (0.09)		8.70b (0.05)
-0.4	6.73c (0.15)		6.19c (0.29)
-0.6	9.80b (0.53)		3.51d (0.31)
-0.8	9.59b (0.77)		0.80e (0.13)
-1.0	14.69a (0.43)		0.21f (0.03)
-1.2	-^a^		-^a^
Source	D.F.	MGT	GI
OP	5	< 0.001***	< 0.001***

Note: All values are showed as mean (S.E.) (*n* = 8). Within a column for MGT and GI of each temperature, values followed by different letters are significantly different by least significant differences of Duncan’s new multiple range test.

*** means *P* < 0.001.

^a^, seed were no germination at -1.2 MPa; thus, MGT and GI could not be calculated.

### pH

**Fig 5:**
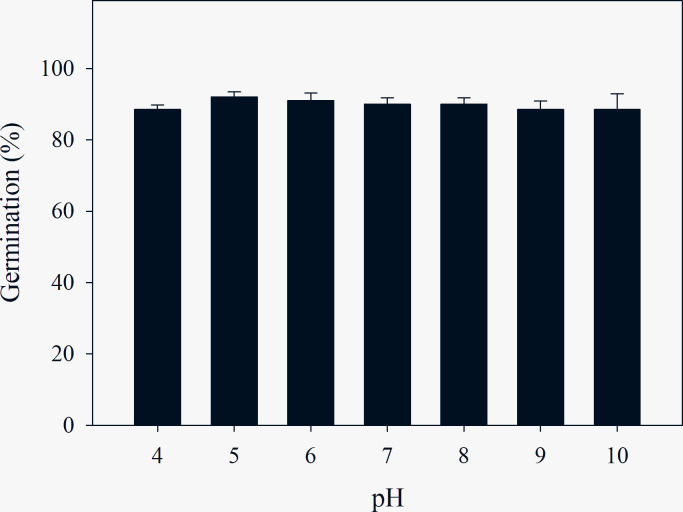
Effect of pH on germination percentage of *C*. *album*. Data represent the mean ± standard error of the mean (*n* = 8).

A moderate *C*. *album* seed germination response was observed across all pH values (>88%) for seeds, with little variation (maximum germination percentage, 92% at pH 5; minimum germination percentage, 88% at pH 9–10) (Fig 5). Germination was relatively consistent across the entire pH range, indicating that this species may germinate readily across a wide range of soil types. Across different pH levels, the MGT was also only slightly affected (Table 5). The maximum MGT of *C*. *album* (4.1 d) was recorded at pH 4–5 and pH 10, while the minimum MGT (3.8 d) occurred at pH 6–7.

**Table 5:** Effect of pH on germination traits of *C*. *album*.

pH	Mean germination time (MGT) (days)		Germination index (GI)
4	3.93abc (0.08)		11.85a (0.10)
5	4.09a (0.01)		11.87a (0.18)
6	3.85bc (0.02)		12.42a (0.34)
7	3.76c (0.06)		12.65a (0.20)
8	4.01ab (0.07)		11.82a (0.34)
9	3.88bc (0.05)		12.09a (0.52)
10	3.99ab (0.08)		11.75a (0.67)
Source	D.F.	MGT	GI
pH	6	0.018*	0.576 ns

Note: All values are showed as mean (S.E.) (*n* = 8). Within a column for MGT and GI of each temperature, values followed by different letters are significantly different by least significant differences of Duncan’s new multiple range test. * means *P* < 0.05 and ns mean no significance.

### Burial depth

**Fig 6:**
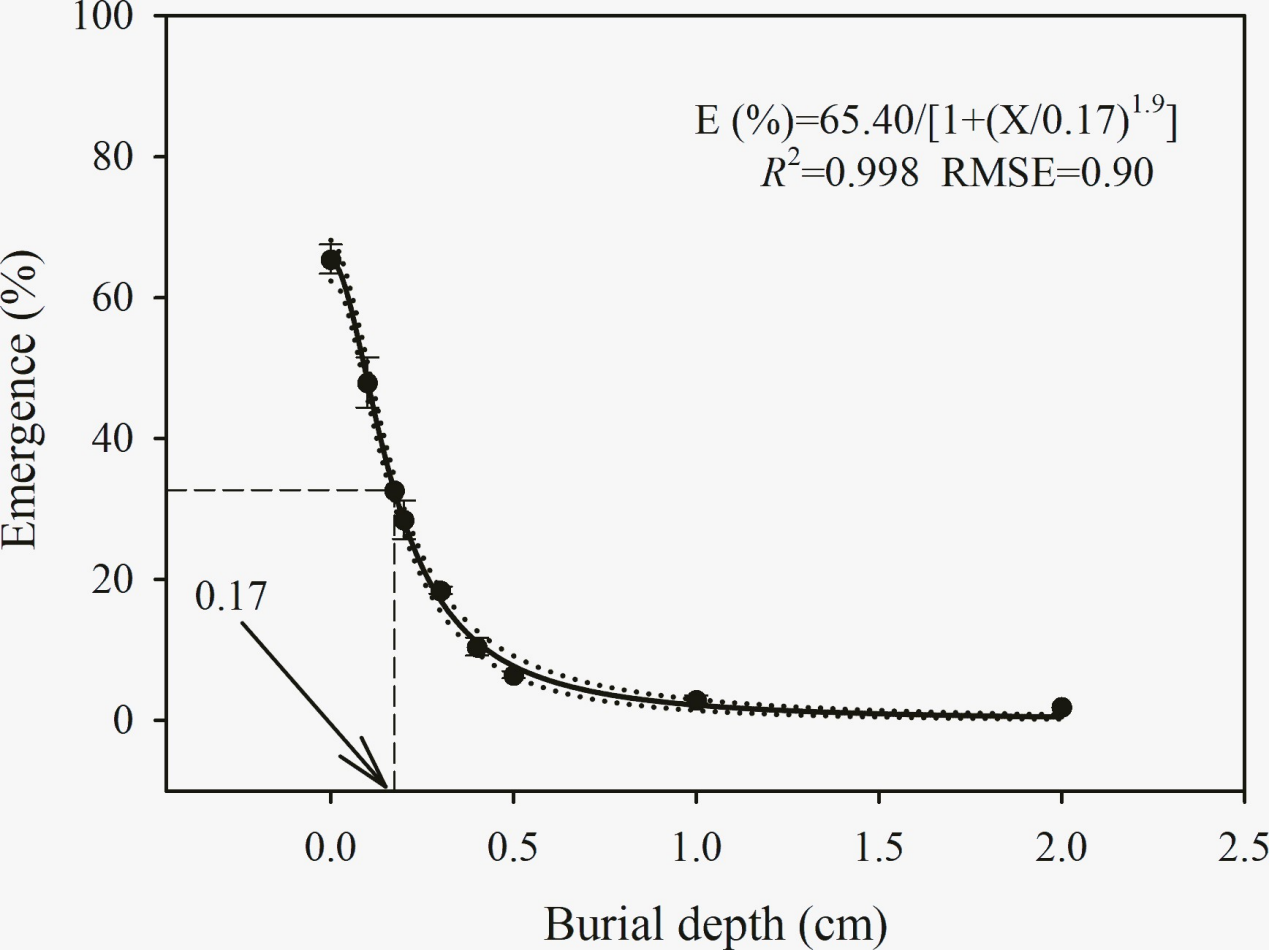
Effect of soil burial depth (cm) on seedling emergence percentage of *C*. *album*. The solid lines represent a three-parameter logistic model fitted to the data of *C*. *album* and thin dotted lines show 95% confidence intervals. E (%) = 65.4/[1 + (x/0.17)^1.9^], *R*^2^ = 0.998, RMSE = 0.90. Data represent the mean ± standard error of the mean (*n* = 8).

A three-parameter logistic model {*E* (%) = 65.4/[1 + (*x*/0.17)^1.9^], *R*^2^ = 0.998, RMSE = 0.90} was fitted to the observations of *C*. *album* seedling emergence across a range of burial depths (Fig 6). Seedling emergence of *C*. *album* decreased with burial depth and decreased from 65% to 2% as the burial depth increased from 0 to 2 cm. According to the model, suppression of 50% of emergence would be achieved at a depth of 0.17 cm (Fig 6).

The MEG and EI values of *C*. *album* seedling emergence were significantly affected by burial depth. The minimum MEG of *C*. *album* was recorded under burial at the soil surface (0 cm) while the highest MEG of *C*. *album* was observed under a burial depth of 2 cm (Table 6). The maximum EI (4.45) of *C*. *album* was calculated at the soil surface, whereas the minimum EI (0.05) was recorded at a 2 cm burial depth. Seed germination and seedling emergence data indicated a strong preference for germination on the soil surface or emergence from a very shallow burial depth (less than 0.5 cm) (Fig 6).

**Table 6:** Effect of soil burial depth on seedling emergence traits of *C*. *album*.

Burial depth (BD) (cm)	Mean germination time (MGT) (days)		Germination index (GI)
0	9.11d (0.45)		4.45a (0.15)
0.1	10.79cd (0.24)		2.77b (0.24)
0.2	12.53bc (0.96)		1.50c (0.12)
0.3	14.44b (1.06)		0.93d (0.09)
0.4	11.20cd (1.17)		0.54e (0.03)
0.5	9.46d (0.39)		0.35ef (0.03)
1	11.50cd (0.50)		0.13f (0.02)
2	18.33a (0.33)		0.05f (0.00)
Source	D.F.	MGT	GI
BD	7	0.007**	0.006**

Note: All values are showed as mean (S.E.) (*n* = 8). Within a column for MGT and GI of each temperature, values followed by different letters are significantly different by least significant differences of Duncan’s new multiple range test.

** means *P* < 0.01.

### Mulch cover

**Fig 7:**
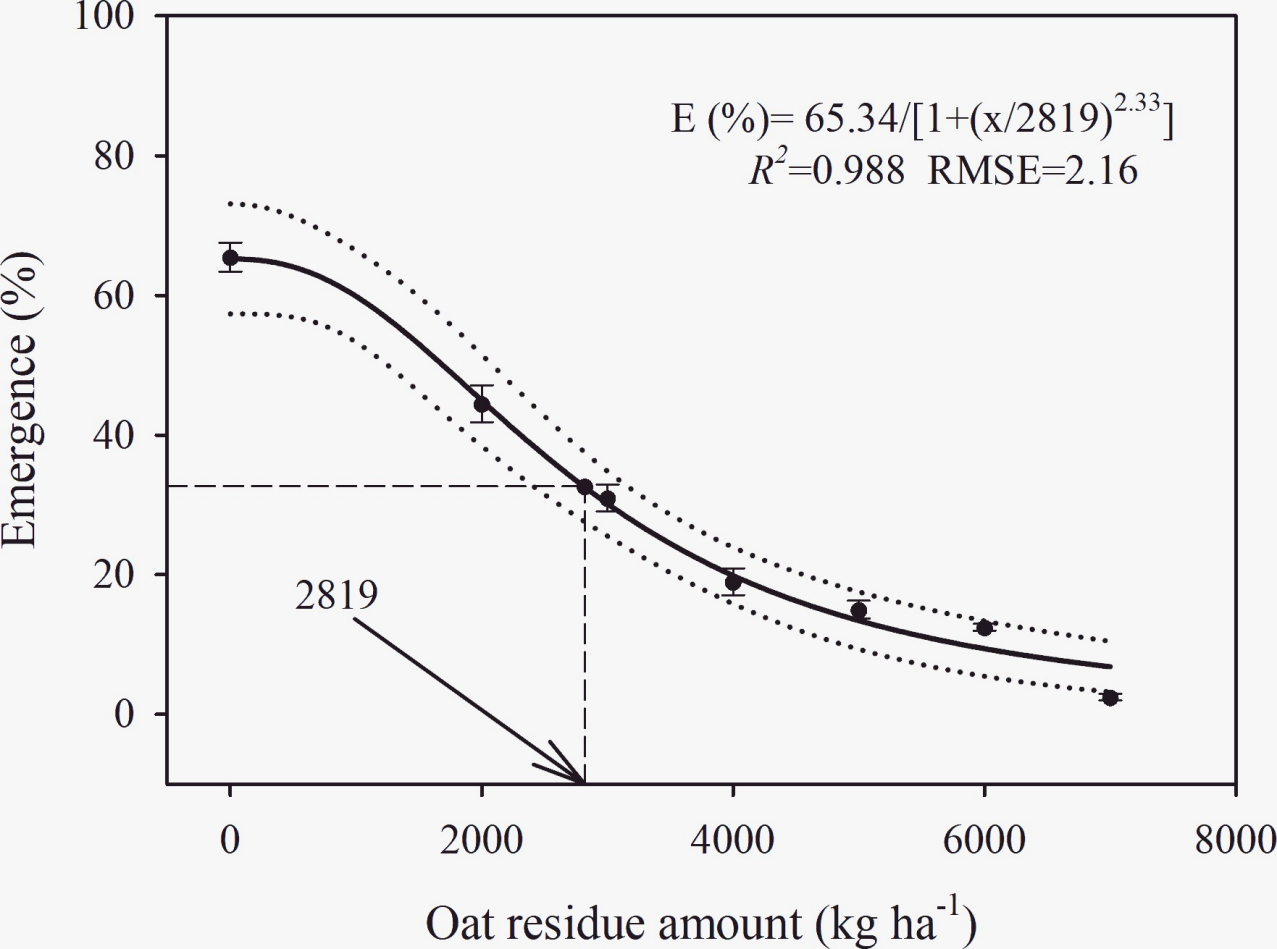
Effect of oat mulch doses (kg/ha) on seedling emergence percentage of *C*. *album*. The solid lines represent a three-parameter logistic model fitted to the data of *C*. *album* and thin dotted lines show 95% confidence intervals. E (%) = 65.3/[1+ (x/2819)^2.3^], *R*^2^ = 0.988, RMSE = 2.16. Data represent the mean ± standard error of the mean (*n* = 8).

A three-parameter logistic model {*E* (%) = 65.3/[1+ (x/2819)^2.3^], *R*^2^ = 0.988, RMSE = 2.16} was fitted to the observed *C*. *album* seedling emergence data across mulch cover treatments (Fig 7). The different application levels of mulches strongly mediated *C*. *album* seedling emergence. A constant reduction in seedling emergence of *C*. *album* was observed as mulch application increased. Seedling emergence was almost entirely depressed under 7000 kg ha^-1^ mulch application (2.5%). Based on the model, the mulch application level required to stop 50% of the final seedling emergence percentage was 2819 kg ha^-1^ (Fig 7).

The MEG and EI of *C*. *album* seedling emergence were significantly affected by mulch application level (Table 7). The minimum MET (9.1 d) of *C*. *album* was recorded under the 0 kg ha^-1^ mulch application, while the highest time to mean emergence (11.3 d) was observed under 6000 kg ha^-1^ mulch application. The maximum EI (4.4) of *C*. *album* was observed under 0 kg ha^-1^ mulch application, while the minimum EI (0.11) was recorded under 7000 kg ha^-1^ mulch application. Generally, the EI value decreased with increased application of mulch.

**Table 7:** Effect of oat mulch doses on seedling emergence traits of *C*. *album*.

Mulch (kg ha^-1^)	Mean germination time (MGT) (days)		Germination index (GI)
0	9.11b (0.45)		4.45a (0.15)
2000	9.69ab (0.47)		2.67b (0.23)
3000	10.18ab (0.50)		1.93c (0.12)
4000	10.66ab (0.19)		1.12d (0.06)
5000	10.71ab (0.44)		0.79d (0.10)
6000	11.27a (0.62)		0.63d (0.04)
7000	10.88ab (0.43)		0.12e (0.03)
Source	D.F.	MGT	GI
Mulch	6	0.044*	<0.001***

Note: All values are showed as mean (S.E.) (*n* = 8). Within a column for MGT and GI of each temperature, values followed by different letters are significantly different by least significant differences of Duncan’s new multiple range test.

* and *** mean *P* < 0.05 and *P* < 0.001, respectively.

## Discussion

*C*. *album* has widely infested many agricultural soils, and it causes substantial interference with crop productivity (e.g., decreased yield and quality), thereby threatening food security broadly [7, 8]. A primary reason for its flexible adaptation relies on higher seed germination potential under benign and adverse environmental conditions [22]. Furthermore, seed germination ability further warrants range expansion ability to the areas facing different types of abiotic stress, including salinity and drought [1]. In this study, the results showed *C*. *album* was able to germinate under diverse environments, indicating that *C*. *album* have a wide seed germination niche. Understanding seed germination ecology can facilitate a comprehensive IWM program to address *C*. *album* infestation in cropping system [7]. The implications of the results are considered below.

### The effect of photoperiod on germination

Rare germination was recorded under continuous dark conditions, demonstrating that *C*. *album* seeds are highly reliant on light to stimulate germination. Thus, the light-dependent *C*. *album* exhibit more viable seeds germination and subsequent seedling emergence when seeds are at the soil surface after seed dispersal in the field [22]. Chamkhi [8] and Alshallash [26] indicated that light could be a prerequisite for germination of this species, which exhibited rare germination in darkness across all temperatures tested. The light environment of the seeds provides essential information that triggers dormancy termination and the subsequent germination of many weed seeds in the proper environmental situation [41]. Consistent with our study, some weed species are known to have positively photoblastic seeds, including *Caucalis platycarpos* L. [42] and *Cyperus aromaticus* (Ridl.) Mattf. & Kuek. [43]. This suggests that seed germination can occur in the upper layer of the soil surface under light stimulation [32]. However, for other species, light is not a prerequisite for germination. In contrast to *C*. *album*, germination is not inhibited in *Achnatherum inebrians* (Hance.) Keng. [44] or is only slightly inhibited in *Myagrum perfoliatum* L. [45] in complete darkness. Thus, these species may germinate under shade or when buried at a shallow depth in soil. Yet, under complete darkness, germination of *C*. *album* seeds was only 13.5%, which may prevent ultimately fatal germination of deeply buried seeds in soil. In turn, dark-induced inhibition of *C*. *album* seed germination would increase the seedbank life [22].

### The effect of temperature on germination

The results obtained from our study showed that *C*. *album* is able to germinate over a range of temperatures (15−40°C), indicating such temperature adaptation may provide *C*. *album* with competitive advantages throughout crop growing seasons across diverse regions. Low GI and delayed MGT of *C*. *album* was observed with increases or decreases from the optimum temperature (25–30°C), indicating that the effective accumulated temperature is reached more quickly at an optimum temperature than at other temperatures. Previous findings have indicated that the optimum temperature for seed germination of *C*. *album* is relatively high (15–27.5°C) [13, 29]. Low-temperature stress had a greater inhibition effect on germination than high-temperature stress, suggesting that this species could be less problematic during cold seasons or at higher altitudes in both temperate and tropical regions [1]. Reduced germination with decreased temperature has also been reported in other *Chenopodium* species, including *C*. *aristatum* and *C*. *pallidicaule* [23, 46, 47]. In this study, the failure of *C*. *album* seeds to germinate in the lowest temperatures suggests temperature-induced dormancy occurs during winter. Meanwhile, after-ripening occurs during late autumn and winter, which results in a large percentage of seeds becoming non-dormant in spring, thus improving *C*. *album* seed germination [12]. Therefore, *C*. *album* seeds retain the ability to germinate at temperatures that occur in the field during late spring and summer [8]. Further investigations of the possibility of a non-dormancy seasonal temperature regime for *C*. *album* will inform management decisions for control of this weed under future climate change scenarios.

### Effect of salt concentration on germination

Seed germination was inversely proportional to salinity for *C*. *album*. GI decreased with increasing salinity, whereas the reverse was true for MGT. Our data indicated that *C*. *album* showed tolerance to salt stress up to 250 mM NaCl. Similar salt-tolerance of *C*. *album* seed germination was observed earlier by Le et al. [21], who indicated that 10% germination occurred at 320 mM NaCl. The slight differences among populations in their sensitivity to salt stress could be owing to intense selection on seeds across different environments. Our results are also similar to those in other *Chenopodium* species, including *C*. *quinoa*; It was previously found that *C*. *quinoa* seeds could germinate and start growing under relatively high salinity in 200 mM NaCl and 40% seawater [48]. Salinity influences seed germination by imposing osmotic stress, which limits water uptake by seeds, resulting in insufficient moisture to start the internal biochemical processes [49]. In addition, the accumulation of toxic ions within cells though an increase in the absorption of toxic substances during the process of imbibition could disrupt the physiological metabolic activity required for germination [50]. Seeds of *C*. *album* are likely to germinate in highly saline soil conditions; for example, regions with high levels of soil salinity fed by brackish/saline water in coastal areas are common in some parts of China [51]. Our study suggests that this potential of *C*. *album* to germinate under moderate to high salinity conditions could allow it to become an increasingly aggressive competitor to field crops in saline environments. Furthermore, precipitation, changes in water distribution, or irrigation water with very low salinity could promote establishment of this species, its spread across diverse cropping systems, and its emergence as an invasive species in the future [21]. Therefore, *C*. *album* could compete with crops in cropping systems with high levels of salinity through its resilience to this abiotic stress.

### Effect of osmotic potential on germination

In the current study, increased osmotic potential exerted a negative effect on *C*. *album* seed germination parameters (e.g., decreased GP and GI and delayed MGT), indicating that the spread of *C*. *album* would most likely be facilitated in soils with higher moisture as well as areas with sufficient precipitation. Water availability is required for imbibition and activation of metabolic reactions, which regulate embryo development and seedling growth [52]. Seeds cannot reach the critical moisture threshold required for imbibition under osmotic stress conditions and thus fail to germinate [40]. In this respect, reduced germination could serve as a survival strategy for unpredictable and low precipitation conditions, which guarantees seed longevity within seedbank until sufficient moisture is available for seed germination [42]. High *C*. *album* germination in the treatment without water stress suggests that a uniform distribution of rains throughout the year in the southeast and southwest of China and irrigated cropping systems could favor the spread of this weed. The high *C*. *album* germination percentage under relatively dry conditions (−0.6 MPa) confirms that it can survive in dryland agriculture systems, although the species would still favor additional surface soil moisture in conservation tillage systems [53]. Our results agreed with those of Šoštarčić et al. [28], who found that lowest water potential for the germination of *C*. *album* occurred under −1.38 MPa [22°C, 12 h/12 h (day/night)]. This suggests that the drier conditions anticipated under climate change would enhance the potential for germination of *C*. *album* globally. Our results are also similar to those obtained in other *Chenopodium* species, including *C*. *quinoa*. Germination of *C*. *quinoa* seed decreased from 75% to 35% with a decreased osmotic potential concentration from 0 to −0.8 MPa [54], and no *C*. *glaucum* seed germinated under −1.2 Mpa PEG treatment or stronger treatments [55]. Thus, based on our observations, the spread of *C*. *album* would most likely be restricted to soils with higher moisture, rather than areas of low precipitation. *C*. *album* germination inhibition under severe drought stress might induce seed dormancy within the soil seedbank, which could constitute an important survival mechanism, safeguarding seedlings from drought conditions until sufficient water is available for successful seedling emergence and establishment.

### Effect of pH on germination

Overall, pH did not have any significant effect on germination of *C*. *album* seeds, which exhibited at least 88% germination regardless of the pH level tested. The capability of this weed to tolerate an extensive range of pH conditions presents a significant fitness and competitive advantage across diverse habitats. Thus, the adaptability of *C*. *album* to germinate in a wide range of soil pH conditions indeed corresponds to most soils in China [56, 57]. This trait of *C*. *album*, common to many weed species, allows it to exploit both acidic and basic soil environments [58]. There is relevant literature on another weedy *Chenopodium* species, e.g., *C*. *glaucum*. The observations of Gulnar et al. [59] in *C*. *glaucum* were similar to those of the present study, with an average germination above 85% at pH 7.2 to 10.7. Thus, the comprehensive adaptability of *C*. *album* to germinate across a wide range of pH values strongly suggests that soil pH is not a constraint to the dispersal or establishment of *C*. *album* across different habitats.

### Effect of seed burial depth on emergence

*C*. *album* seedling emergence initially decreased and was delayed as soil depth increased. The negative correlation between emergence and burial depth is consistent with positively photoblastic seeds, as less than 0.01% of the incident light could be penetrated to a soil layer depth of 4 mm in sandy soil or 1 mm in dark soil [60, 61]. Thus, a light requirement for germination could acts as a surface-sensing mechanism, enabling small *C*. *album* seeds to germinate when found near the soil surface. In contrast, buried seeds from depths, which they are unable to emerge physically, would remain dormant and germinate at a subsequent stage, after soil disturbance [7, 61]. In addition, lower seedling emergence at deeper burial depths also might be the consequence of selection on gas exchange and seed nutrient reserves [60]. When seeds are buried, there is limited gas exchange through the seed coat, thereby inhibiting germination [62]. Moreover, given that *C*. *album* is a small-seeded species, its newly germinated seedlings lack carbohydrate reserves for the coleoptiles to push through the soil layers, resulting in a failure to emerge [21]. Consequently, these mechanisms of emergence inhibition may be important strategies that preserve the *C*. *album* soil seedbank [63]. Further research is required to investigate the length of *C*. *album* seed viability in the seedbank after burial, which is of great importance for effective weed management.

Our results are similar to those of a previous study conducted in Korea, in which almost all *C*. *album* seeds germinated at a burial depth of 0.5 cm in horticultural nursery soil, and increased burial depth decreased seedling emergence, with no seedlings emerging from seeds at a 4 cm burial depth [21]. The emergence differences in sensitivity to burial depth could be mediated by soil type, as light transmittance decreases with decreasing soil particle size and increases with the content of darker components (e.g., soil organic matter) [61]. Similar to our findings, *C*. *pallidicaule*, a non-weedy *Chenopodium* species, exhibited maximum seedling emergence at a 0.5 cm depth, and only few seedlings emerged when seeds were buried at a 5 cm depth [47]. One possible reason for the higher germination at relatively shallow burial depths may be higher soil-seed contact. The results obtained in our study suggest that small-seeded species like *C*. *album* appear to preferentially emerge on the soil surface or from very shallow burial depths, which would thus yield the highest survival under a natural, no-tillage system. Thus, a shallow-tillage operation, with seeds buried at least below the depth of emergence in infested areas, could be an IWM approach to control *C*. *album* in agricultural systems. Meanwhile, tillage techniques that may bring buried seeds back to the soil surface should be avoided.

### Effect of mulch cover on emergence

The use of crop residue is popular in the wake of the movement toward sustainable agriculture to suppress the germination and growth of weeds [64]. The application of crop residue on the soil surface might create a physical obstruction and shading effect that decreases the emergence of weeds [65]. In the current study, seedling emergence percentage and EI of *C*. *album* significantly decreased as the amount of oat residue placed on the soil surface increased, whereas the reverse pattern was observed for MET. From our light experiment, it was also evident that the complete-dark condition reduced the germination of *C*. *album* seeds. Thus, this study suggests that a crop residue amount of 6000–7000 kg ha^−1^ (commonly observed in fields) would create a shade cover and physical barrier in the field, thereby decreasing *C*. *album* emergence. In agreement with our results, Loura et al. [4] found that the application of sorghum residue at a level of 6,000 kg ha^-1^ reduced emergence of *Conyza bonariensis* L. Cronquist by 56.5%, compared with a no-residue treatment. Similarly, Amini et al. [66] also reported that wheat residue cover (5000 kg ha^−1^) decreased the seedling emergence of *Astrodaucus orientalis* (L.) Drude by 80%, compared with no residue cover treatment. Furthermore, it has also been found that low seedling emergence and seedling growth of the weed *Hypericum triquetrifolium* Turra could also be caused by allelochemicals and other allelopathic products of oat [67], but the effect of allelochemicals was not specifically evaluated in our study.

In the present study, chopped oat residue was spread evenly in pots, whereas crop residue might be intact in the field. Moreover, the highest oat residue amount (7000 kg ha^-1^) did not completely suppress *C*. *album* seedling emergence. Therefore, it is possible that there is a need for IWM practices in which residue cover is employed together with other control options for effective *C*. *album* management in agricultural habitats. Overall, this study suggests that crop residue retention or mulch as a component of conservation-tillage practices could help in controlling *C*. *album*.

## Conclusion

The remarkable ability of *C*. *album* to germinate under a wide range of environmental conditions and its abiotic stress tolerance enables it to infest diverse agricultural soils. These robust seed biology features could provide the species with a greater capacity to interfere with crop productivity in many agricultural fields in various parts of the world. Abundant water can promote germination in spring, summer, and autumn, but germination is unlikely to be significant under low soil moisture and high salinity. Germination is also likely under a wide range of soil pH values, which is not a limiting factor for the germination of this species. Light increases germination of *C*. *album* seed; therefore, the *C*. *album* possibly persists in no-till agricultural systems, as the seeds will readily germinate if allowed to remain on the soil surface after seed shed. Hence, by depriving the seeds of light using shallow tillage, it would bury most *C*. *album* seeds below their maximum depth of emergence resulting in reducing the occurrence of seedbank build-up on the soil surface. Alternatively, mulch cover practices can create a shading effect and physical barrier in the field, thereby suppressing *C*. *album* emergence in no-till agricultural systems. Thus, shallow tillage operation and the retention of crop residue cover on the soil surface would be an integrated management approach for establishing a crop in fields infested with of *C*. *album* in conservation agriculture systems. Furthermore, management of this weed would also require approaches that minimize soil seedbank input. Therefore, approaches to reduce the seed reserves to a negligible level for reestablishment to occur, in particular, should be studied further.
